# Disposable impedance sensors based on novel hybrid MoS_2_ nanosheets and microparticles to detect *Escherichia Coli* DNA

**DOI:** 10.1371/journal.pone.0299272

**Published:** 2024-02-29

**Authors:** Tien Ngoc Phuc Nguyen, Son Hai Nguyen, Mai Thi Tran

**Affiliations:** 1 College of Engineering and Computer Science, VinUniversity, Hanoi, Vietnam; 2 VinUni-Illinois Smart Health Center, VinUniversity, Hanoi, Vietnam; 3 School of Mechanical Engineering, Hanoi University of Science and Technology, Hanoi, Vietnam; Chulalongkorn University Faculty of Engineering, THAILAND

## Abstract

The rapid and accurate detection of pathogenic bacteria is essential for food safety and public health. Conventional detection techniques, such as nucleic acid sequence-based amplification and polymerase chain reaction, are time-consuming and require specialized equipment and trained personnel. Here, we present quick, disposable impedance sensors based on the novel hybrid MoS_2_ nanomaterial for detecting *Escherichia coli* DNA. Our results indicate that the proposed sensors operate linearly between 10^- 20^ and 10^−15^ M concentrations, achieving an impressive detection limit of 10^−20^ M with the highest sensitivity observed at a 0.325 nM probe concentration sensor. Furthermore, the electrochemical impedance spectroscopy biosensors exhibited potential selectivity for *Escherichia coli* DNA over *Bacillus subtilis* and *Vibrio proteolyticus* DNA sequences. The findings offer a promising avenue for efficient and precise DNA detection, with potential implications for broader biotechnology and medical diagnostics applications.

## Introduction

In recent decades, foodborne and waterborne diseases have become an increasing threat to global health, resulting in severe infectious diseases and causing morbidity or even mortality in different locations [1]. Statistics provided by the World Health Organization (WHO) reveal that foodborne illnesses cause the deaths of 420,000 people each year worldwide, 30% of whom are children under five years old. Meanwhile, waterborne diseases are responsible for the deaths of approximately 1.6 million people annually, affecting both developing and developed countries [2]. These issues primarily stem from the toxins and infection mechanisms of pathogens in the environment. *Escherichia coli* is one of the most prevalent pathogenic bacteria infected through contaminated food ingestion and water [3]. *E*. *coli* is primarily a commensal bacterium found in the intestines of mammals, including humans [4]. It can also be found in the external environment, particularly in animal waste, wastewater, dairy products, and uncooked food [5]. While most strains of *E*. *coli* are harmless in the intestines, certain toxic strains can cause illnesses ranging from moderate symptoms such as stomach cramps, vomiting, and diarrhea, to severe conditions like sudden kidney failure [4]. Therefore, there is a significant demand for effective methods to detect E. coli in order to monitor food and water safety and protect global health.

Culturing, the conventional procedure for detecting *E*. *coli*, is typically time-consuming and requires a laboratory [6]. Nucleic acid-based (e.g., Polymerase Chain Reaction, Nucleic Acid Sequence-Based Amplification, Loop-Mediated Isothermal Amplification, and microarray technology) and immunologically based (e.g., Enzyme-Linked Immunosorbent Assay and Lateral Flow Immunoassay) techniques offer faster detection times, notable sensitivity, and specificity [7]. Even though these assays improve time efficiency, they are not optimal due to their limited detection range, incompatibility with laboratory equipment, and high cost [8]. Hence, the biosensor-based method has recently acquired popularity due to its ability to overcome these obstacles [8]. In particular, electrochemical biosensors have recently emerged as a potential candidate for a point-of-care and non-laboratory DNA detection device with substantially high sensitivity, specificity, and stability while ensuring fast response time and cost-effectiveness [8, 9]. Interdigitated electrodes are commonly used as transducers in these electrochemical sensors due to their superior sensitivity, high surface area, rapid response, and low cost [10, 11]. Additionally, it is widely accepted that probes can enhance the selectivity and sensitivity of DNA biosensors [12]. Researchers have developed a probe as the binding element between the sensing nanomaterial and the targeted analyte in the biosensor system.

Two-dimensional (2D) MoS_2_ materials have garnered interest in the field of electrochemical biosensing due to their outstanding electrical properties, such as changing electronic energy states, offering a relatively narrow band gap, and high electron mobility [13–15]. Furthermore, the capabilities of MoS_2_ can be enhanced by combining it with other nanomaterials or even its precursors to form a new hybrid structure. This combination can overcome some limitations of pure nanomaterials by increasing the electrical conductivity, surface area, and stability, thereby meeting specific needs with improved performance [16]. These advantages have motivated our research into hybrid MoS_2_ materials for developing a label-free, real-time, disposable, cost-effective impedance biosensor that electrochemically detects the DNA of pathogenic bacteria.

This paper introduces a new sensing platform utilizing hybrid MoS_2_ nano/microstructures and an NH_2_ probe sensor. This platform employs the electrochemical impedance spectroscopy (EIS) technique to detect *E*. *coli* DNA concentrations ranging from 10^−20^ to 10^−15^ M. To the best of our knowledge, there are no existing reports on the use of novel hybrid-MoS_2_ materials for *E*. *coli* DNA detection via impedance sensors. Our findings will provide valuable insights for researchers focused on pathogen detection applications involving hybrid 2D MoS_2_ materials.

## Methods and materials

### Chemicals and probe

The chemicals used in this research without further purification were Ammonium Heptamolybdate Tetrahydrate ((NH_4_)_6_Mo_7_O_24_.4H_2_O, 99.0%, Tianjin Chemical Reagent Factory, Tianjin, China), https://pubchem.ncbi.nlm.nih.gov/compound/ThioacetamideThioacetamide (C_2_H_5_NS, 99.0%, Shanghai Zhanyun Chemical Co., Ltd, Shanghai, China), Ethanol (C_2_H_5_OH, 99.5%, Xilong Scientific Co., Ltd., Guangdong, China), and deionized (DI) water.

The oligonucleotide probe was designed to target *E*. *coli* using the sequence amino-5′-GGTCCGCTTGCT CTC GC-3′ [17]. *E*. *coli*, *Vibrio proteolyticus*, and *Bacillus subtilis* DNAs were pretreated by heating at 95°C for 30 minutes during the experiments.

### Hydrothermal method to prepare hybrid MoS_2_ nanomaterials

Hybrid-type MoS_2_ nanosheets were prepared using the hydrothermal method [18]. The process was as follows. First, 5 grams of (NH_4_)_6_Mo_7_O_24_.4H_2_O and C_2_H_5_NS were completely dissolved in 20 mL of DI water and stirred separately for 10 minutes. These solutions were then combined and stirred together for an additional 5 minutes. Next, 20 mL of Ethanol was gradually added to the mixture, which was then stirred for 30 minutes. The resulting precipitate was transferred to an 80 mL Teflon-lined stainless-steel autoclave, kept at 180°C for 10 hours, and allowed to cool naturally to room temperature. Finally, the products were collected by centrifugation at 5000 rpm for 4 minutes, washed three times with DI water and Ethanol, and then dried in a vacuum at 60°C for three hours.

### Electrochemical impedance spectroscopy setup and measurements

The interdigitated electrodes (IDE) feature a comb-like shape with 20 fingers, a gap size of 200 μm, and a finger’s width of 400 μm [19]. These electrodes are made of aluminum coated with lead. This design is simple, quick, and low-cost to prepare. A significant advantage of the interdigitated electrodes is that they operate without the need for a redox electrode and the labeling of the sensing film [20]. The IDE-based sensor functions as an electrochemical transducer, detecting the slight variation in resistance or capacitance of various analytes. In our study, we use electrochemical impedance spectroscopy to observe the behaviors of the sensor and quantitatively measure the electrical signals corresponding to *E*. *coli* DNA concentrations at various frequencies.

**Fig 1:**
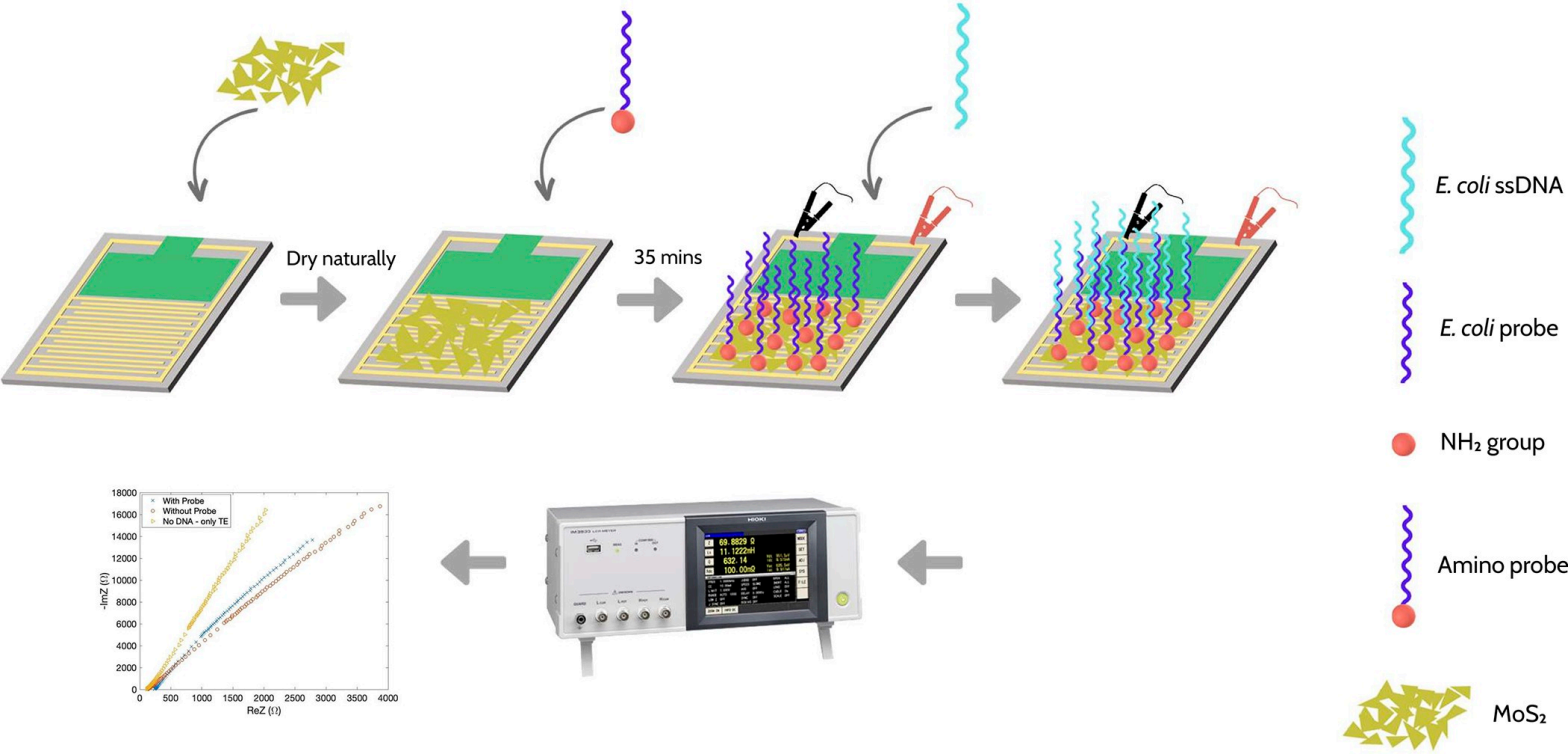
The experimental procedure of *E*. *coli* DNA detection based on hybrid MoS_2_ nanomaterials IDE sensors.

Our experimental procedure is shown in Fig 1. First, the electrodes were cleaned with Ethanol and then spin-coated with 100 μL of sensing nanomaterials. The film was dried at room temperature for a few hours. Next, we added a 100 μL solution of a 32.5 nM amine probe on the surface of the sensing thin film. The probe NH_2_-5′-GGTCCGCTTGCT CTC GC-3′ was selected to detect the complementary target *E*. *coli* DNA according to the Watson-Crick base-pairing rules. This probe was modified with amine (-NH_2_) to bond with the hybrid MoS_2_ surface. After 40 minutes of incubation, we gradually added *E*. *coli* DNA with different concentrations. Electrochemical characterization was conducted using the Hioki LCR IM3536. The DC voltage was set to 10 mV to ensure operation within the linear working domain. The frequency was scanned from 4 Hz to 1 kHz. The impedance and the phase difference data were acquired to explore the chemical binding effects of *E*. *coli* DNA on the electrode surfaces in real-time.

### DNA extraction

The *E*. *coli*, *B*. *subtilis*, and *V*. *proteolyticus* bacteria in our study were provided by the Microbiology and Genetics Lab at Hanoi University of Science and Technology. The standard procedure for bacterial DNA extraction is as follows. First, a pellet of 1.5 mL of overnight bacteria culture in Luria Broth (LB) medium is centrifuged at 8,000×g for 5 minutes. After discarding the supernatant, the cell pellet in 740 μL of Tris-EDTA (TE) buffer is resuspended. Next, 20 μL of 100 mg/mL Lysozyme is added to degrade the cell wall, followed by 30 minutes of incubation at 37°C. Then, 40 μL of 10% SDS and 8 μL of Proteinase K (10 mg/mL) from Biobasic, Canada, are added to aid protein digestion and membrane disruption. After incubation at 56°C for 3 hours, 100 μL of 5 M NaCl and heated CTAB/NaCl (Merck, Germany) at 65°C are progressively added to precipitate DNA. After 10 minutes at 65°C, the material is extracted with Sigma Aldrich chloroform: isoamyl alcohol to separate DNA from contaminants. After centrifuging at 12,000×g for 10 minutes at room temperature, the DNA-containing aqueous phase is transferred to a fresh tube. This extraction procedure is repeated until no white protein layer remains. The DNA is precipitated with 100% Ethanol (Merck, Germany) and kept at -20°C for 2 hours overnight. Following 15 minutes of 12,000×g centrifugation at 4°C, the DNA pellet is washed with 50 μL of 70% Ethanol to remove contaminants and salts. After drying, the pellet is resuspended in TE buffer for storage. It is recommended to store isolated DNA at -20°C for future usage. All DNA samples in this investigation had OD260/280 ratios around 2.0, indicating high purity.

## Results and discussions

### Structure and morphology of the sensing materials

**Fig 2:**
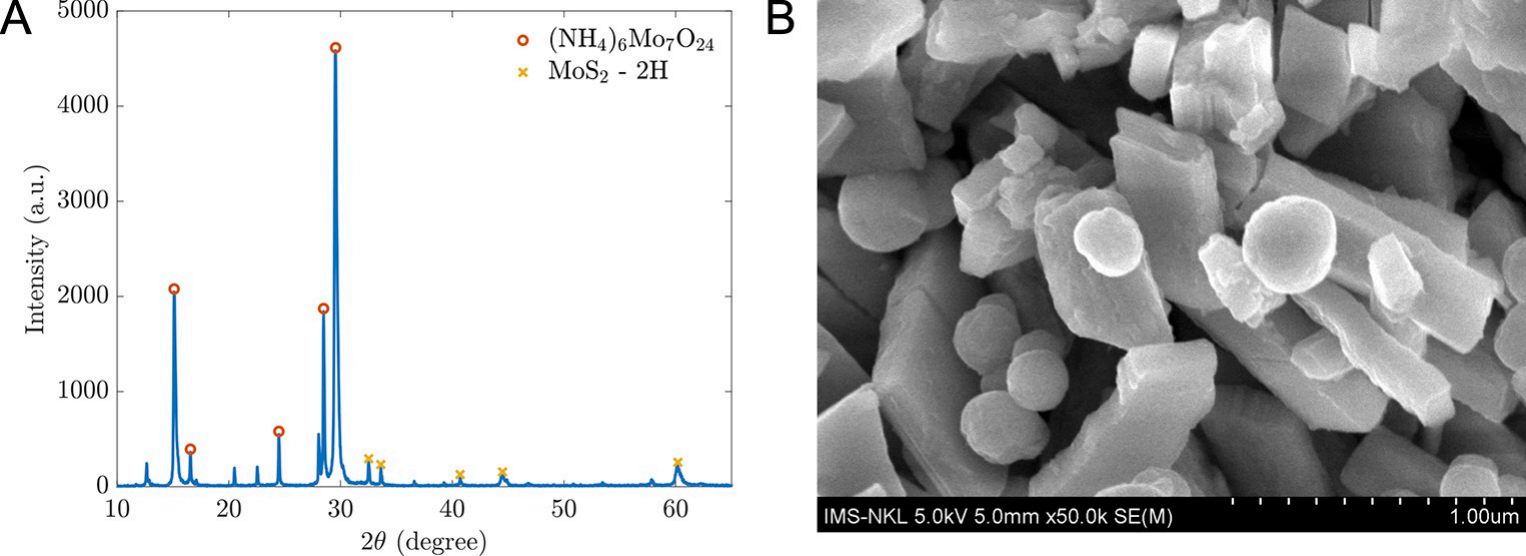
Characterizations of prepared materials. (A) X-ray diffraction spectrum of prepared material taken by Rigaku MiniFlex600; (B) SEM image of hybrid MoS_2_-2H nanomaterials taken with Hitachi S-4800.

The structure and morphology of the synthesized materials were analyzed using XRD and SEM observations. As shown in Fig 2A, the composition of hybrid-type MoS_2_ includes MoS_2_-2H (card no PDF#17–0744) and (NH_4_)_6_Mo_7_O_24_ (PDF#23–0784). The X-ray spectrum of MoS_2_-2H shows the diffraction peaks at (101), (012), (015), (110), and (113) planes, corresponding to the peaks centered at the 2*θ* angles of 33.03°, 34.06°, 41.11°, 58.32°, and 60.50°, respectively (PDF #17–0744, analyzed using JADE software by MDI Materials Data). Because the hydrothermal process happened in a short time, 10 hours at 180°C, along with MoS_2_-2H, the precursor chemical Ammonium Heptamolybdate Tetrahydrate ((NH_4_)_6_Mo_7_O_24_) is still found in the resultant composite. However, the SEM image in Fig 2B reveals that the hybrid material is a mix of multi-layer sheets and microparticles of materials. Hence, we hypothesized that either (NH_4_)_6_Mo_7_O_24_ functionalizes the MoS_2_ surface or (NH_4_)_6_Mo_7_O_24_ molecule fragments.

### Direct detection of *E*. *coli* DNA by EIS measurements

**Fig 3:**
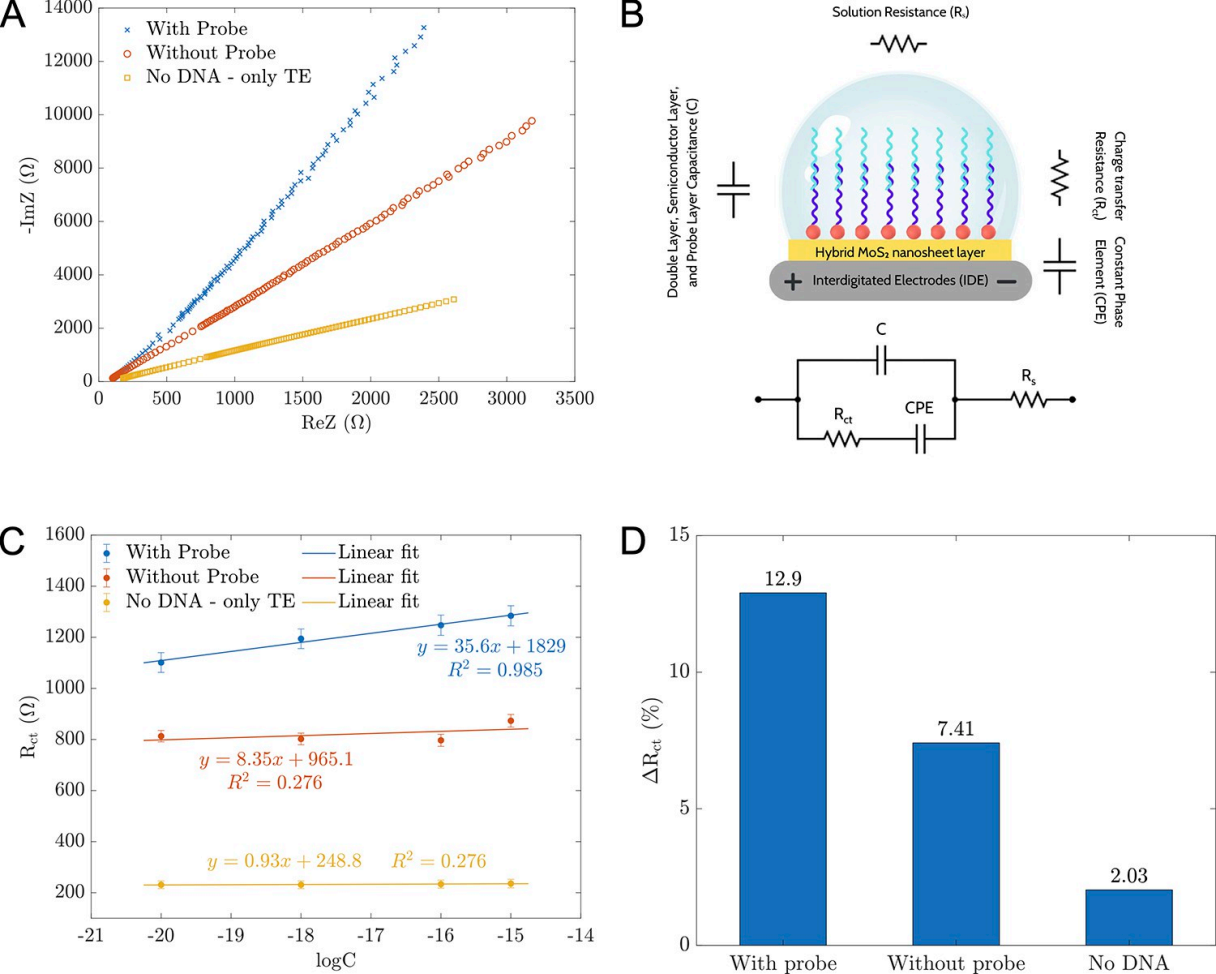
Direct detection of E. coli DNA by EIS measurements. (A) Nyquist plots of impedance from three experiments: proposed sensors in contact with *E*. *coli* DNA, sensors without an amino-probe in contact with *E*. *coli* DNA, and proposed sensors with TE buffer only when adding 10^−16^ M DNA and a TE volume equivalent to 10^−16^ M. (B) The Randles model used to fit the experimental data in this study, and its equivalent physical components in IDE-based sensors. (C) R_ct_ values fitted using the Randles model (Fig 3B) correspond to different analyte concentrations from the three experiments in Fig 3A. Error bars represent the fitting errors from the three data sets. (D) ΔR_ct_ for the three scenarios using Eq (2).

In our experiment, *E*. *coli* DNA concentrations ranging from 10^−20^ to 10^−15^ M were examined, using a 30 μL sample for each concentration level. This range corresponds to 183 to 1.83×10^7^ single-stranded DNA (ssDNA) copies. The impedance responses varied with the *E*. *coli* DNA concentrations or the number of ssDNA, adjusting to the applied frequencies. These responses, reflecting physical events on the IDE surface, are affected by analyte interactions. To evaluate our sensors’ ability to identify *E*. *coli* DNA, we conducted tests using sensors with *E*. *coli* DNA (denoted as “With Probe”), sensors with TE buffer (denoted as “No DNA—only TE”), and sensors with *E*. *coli* DNA but lacking the amine probe (indicated as “Without Probe”). Fig 3A presents the Nyquist plots for 10^−16^ M *E*. *coli* DNA or a corresponding TE volume, illustrating the relationship between the complex impedance Z’s real and imaginary parts. The Nyquist plots from all samples, with concentrations between 10^−20^ to 10^−15^ M, conformed to the Randles model depicted in Fig 3B. The ZView (EIS data analysis software developed by AMETEK Scientific Instruments) was employed for the fitting procedure. This model consistently produced minimal discrepancies across the data sets.

The Randles model integrates capacitors from a probe layer (C) with a constant phase element (CPE) created by a double layer of MoS_2_ hybrid nanomaterials. This CPE, denoting the film’s imperfections, is calculated by Z = 1/(Cjω)^n^, where n ranges between 0 and 1. An n value of 1 signifies an ideal capacitor behavior of the CPE, while 0 indicates pure resistance. Our data consistently showed an n value close to 0.9, suggesting the film’s imperfect smoothness [19]. The equivalent circuit encompasses both the charge transfer resistance (R_ct_) and the solution’s resistance (R_s_). Fig 3B shows the comparative representation of our sensor’s physical components and the components in the circuit model. The charge transfer resistance represents the ability of charges to be transferred between electrodes and was used as an indicator in our proposed sensors. Derived from fitting processes, R_ct_ depends on the logarithm of the DNA concentration (logC), summarized in Table 1 and depicted in Fig 3C and 3D. Only sensors in contact with *E*. *coli* DNA showed a high-precision linear fit with the logarithm of the concentration of *E*. *Coli* DNA (R^2^ = 0.985):

y=35.6x+1820, (1)

where *x* is the logarithm of the concentration of *E*. *Coli* DNA, and *y* is the transfer resistance R_ct_ value (Ohms). Using Eq (1), the unknown DNA concentration level (logC) can be estimated via the fitted R_ct_. Without a probe or in the absence of DNA, the R_ct_ fluctuated within a narrow range and could not be linearly fitted. This result confirmed the effectiveness of our proposed sensors in detecting *E*. *coli* DNA.

**Table 1:** The charge transfer resistances are estimated from the Randles model.

logC ([C] = M)	*E*. *coli*	No DNA	Without Probe
	R_ct_ (Ω)	R_ct_ (Ω)	R_ct_ (Ω)
-20	1101 ± 38.8	231 ± 15.0	812.8 ± 22.3
-18	1194 ± 38.4	231 ± 15.1	802 ± 22.9
-16	1247 ± 39.7	233.5 ± 15.7	796.5 ± 23.6
-15	1284 ± 39.5	235.7 ± 16.8	873 ± 24.6

Fig 3C also reveals a significant variation in the magnitude and linear trend of R_ct_ in different functionalization cases. The R_ct_ value of the sensor coated with hybrid MoS_2_ nanomaterials peaks when both the *E*. *Coli* probe and its complementary single-stranded DNA are immobilized. The R_ct_ value decreases when only *E*. *coli* DNA is present on the sensor’s surface without a probe, and is minimal when there are only probes with no DNA hybridization. For better visualization of the data, the relative difference of R_ct_ values when adding 10^−15^ M and 10^−20^ M was calculated by the following equation:

$$\Delta R_{ct}=100\times \frac{{R_{ct}}_{{(10}^{-15}M)}-{R_{ct}}_{{(10}^{-20}M)}}{{R_{ct}}_{{(10}^{-20}M)}}(\%)$$ (2)

As illustrated in Fig 3D, ΔR_ct_ was only 2.03% when no DNA was presented, increased to 7.41% when no probe was used, and reached 12.9% with our proposed sensors. Two primary factors can account for the variation in charge transfer resistance in each case: (1) the orientation of the probe and bacterial DNA both before and after hybridization and (2) the distribution and alignment of ssDNA within the intermolecular region.

**Fig 4:**
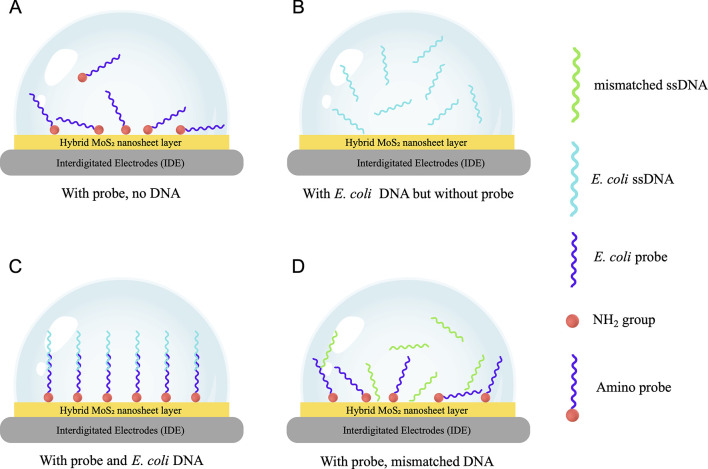
A simple diagram to describe the interaction among the sensor’s elements ssDNA and probe in different cases of functionalization at the working electrode’s surface. Sensors in contact with (A) TE buffer only; (B) hybrid MoS_2_ nanomaterials and *E*. *coli* DNA without a probe; (C) complementary ssDNA from *E*. *coli*; and (D) mismatched ssDNA from *B*. *subtilis* or *V*. *proteolyticus*.

Despite the influence of covalent and hydrogen bonding, the detection mechanism in our sensing platform is mainly based on the electrostatic interaction between DNA molecules and charged elements within the system. The hybrid MoS_2_ nanosheet is a semiconductor nanomaterial with a low band gap. When a voltage is applied to an electrode coated with a layer of hybrid MoS_2_ nanosheet material, the material’s semiconductor properties allow the electrons in the valence band to be excited. These electrons then enter the conduction band and leave behind positively charged hole areas [21]. These positively charged areas can influence the orientation of the DNA probe molecules through electrostatic interaction with the negative charges at their phosphate backbone [22, 23]. When only probe molecules are attached to the sensing platform, they may not align perfectly vertically on the surface. Instead, they could be oriented at various angles due to the attraction of opposite charges on the transducer [24], as illustrated in Fig 4A. When *E*. *coli* ssDNA is present without a probe (Fig 4B), R_ct_ is lower than the first measurement in Fig 4A because the absence of a probe at the contact surface allows charges to move freely between the electrode and the electrolyte at a higher rate, resulting in a decreased R_ct_ value [25].

When both probes and *E*. *coli* DNA are present, as shown in Fig 4C, the R_ct_ is higher and increases with the concentration of DNA. This phenomenon is attributed to the hybridization between probes and *E*. *coli* ssDNA. When the hybridization with complementary DNA happens, the stiff and rod-shaped double-stranded DNA (dsDNA) is formed by the binding of the ssDNA and probe straightens [23]. This process can alter the tilt angle of the DNA molecule relative to the platform’s surface and enhance the net negative charge of the dsDNA molecules. As a result, although the formation of dsDNA may create more space on the surface for electrons to travel through [22], the electrostatic potential among the hybridized DNA chains and around the probe-ssDNA layer might increase. This increase repels nearby electrons attempting to pass through. In addition, the hybridized dsDNA can form a thicker insulating layer than the initial probe film, which significantly hinders the flow of electron transfer at the contact surface between the electrolyte and the sensor. Consequently, there is a significant increase in the R_ct_ of the probe-coated sensor with the introduction of the target DNA [26, 27]. The more hybridizations of the DNA and probe occur, the greater the charge transfer resistance in the system. This explains the linearly increasing trend of R_ct_ when *E*. *Coli* DNA is continuously added in the concentration range from 10^−20^ to 10^−15^ M.

On the other hand, when non-complementary ssDNA from *B*. *subtilis* or *V*. *proteolyticus* is added, hybridization does not happen. The ssDNA molecules distribute randomly on the sensor (Fig 4D), with some lying at various angles on the probe films, some suspended in the solution, and others infiltrating the spaces between the probe molecules. Due to the probe’s ssDNA not straightening as it would during hybridization with complementary *E*. *coli* DNA, fewer spaces are available on the sensor’s surface for electrons to pass through. Moreover, because the net charge of the ssDNA and probe system remains similar regardless of the introduced bacterial DNA, the disordered distribution of mismatched ssDNA may extend the repulsive electrostatic potential region, making it harder for electrons to penetrate and pass through the dense barrier of the like-charged molecules on the sensor surface. Therefore, mismatched DNA presents a greater hindrance to electron flow in a sensor than matched DNA. This assumption will be verified with the experimental data in the next section.

### Selectivity of the sensing platform with two other bacteria

**Fig 5:**
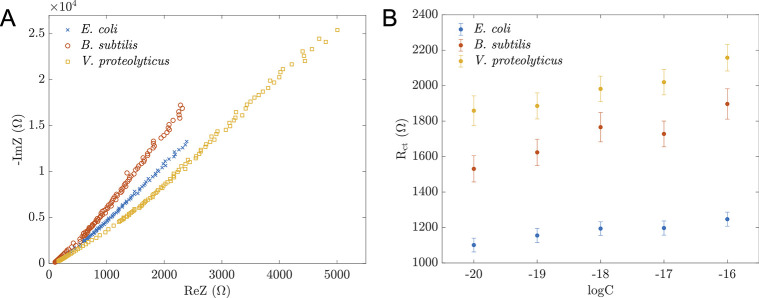
Potential selectivity of proposed sensors. (A) Nyquist plots of impedance from three experiments using proposed sensors when in contact with DNA from *E*. *coli*, *B*. *subtilis*, and *V*. *proteolyticus* at a concentration of 10^−16^ M. (B) R_ct_ values fitted using the Randles model in Fig 3B correspond to different analyte concentrations from the three experiments. Error bars represent the fitting errors from the three data sets.

When examining the selectivity between *E*. *coli* and other bacterial strains, such as *V*. *proteolyticus* and *B*. *subtilis*, our sensing platform shows promising results, as displayed in Table 2 and Fig 5. The sensors exposed to non-complementary DNA from *B*. *subtilis* and *V*. *proteolyticus* exhibit a considerably higher R_ct_ compared to those with *E*. *coli* DNA, demonstrating the ability to distinguish between matched and mismatched DNAs. The R_ct_ of mismatched DNAs was higher than that of *E*. *coli* DNA case. This experimental data agreed with our previously proposed hypothesis. Furthermore, these fitted values of R_ct_ did not change linearly with the DNA concentration. This observation can help discriminate *E*. *coli* and two other DNAs we tested, representing gram-positive and gram-negative bacteria.

**Table 2:** The estimated charge transfer resistances for three types of bacterial DNA.

logC ([C] = M)	*E*. *coli*	*V*. *proteolyticus*	*B*. *subtilis*
	R_ct_ (Ω)	R_ct_ (Ω)	R_ct_ (Ω)
-20	1101 ± 38.8	1859 ± 83.8	1531 ± 74.0
-18	1194 ± 38.4	1982 ± 71.8	1766 ± 82.7
-16	1247 ± 39.7	2158 ± 74.9	1897 ± 85.8
-15	1284 ± 39.5	2217 ± 75.9	1819 ± 70.9

### Effect of probe concentration on the absorption of MoS_2_ biosensors

In the previous section, our experiment showed that the combination of the amino probe and hybrid MoS_2_ nanosheets provides a rapid and highly responsive sensing platform for *E*. *coli* DNA detection based on R_ct_ measurements. We used a probe concentration of 32.5 nM, corresponding to 6×10^11^ copies added to the hybrid MoS_2_ surface. The number of probes was significantly larger than the number of ssDNA in the testing range; the maximum number of ssDNA was 1.8×10^7^, corresponding to a 10^−15^ M test sample. In this section, we investigate the impact of reducing probe concentrations on sensor performance. We prepared two other sensors with probe concentrations of 3.25 nM (equal to 6×10^10^ copies) and 0.325 nM (equivalent to 6×10^9^ copies) and tested them with the same range of *E*. *coli* DNA from 10^−20^ to 10^−15^ M.

Repeating the experiments and analysis, we determined the R_ct_ for each probe concentration. The fitted R_ct_ values linearly increased with the added DNA concentrations, as summarized in Table 3. The R_ct_ values decreased dramatically, and the slope of the calibration line became steeper as the probe concentration decreased tenfold, with the steepest slope being 172 for the 0.325 nM sensors. ΔR_ct_, calculated using Eq (2), showed values of 12.9%, 85.82%, and 158.71% for probe concentrations of 32.5 nM, 3.25 nM, and 0.325 nM, respectively. The change in R_ct_ was less pronounced at higher probe concentrations. This phenomenon may be attributed to more open spaces for electrons to move through because the probe adheres less securely to the sensor’s surface. Therefore, higher probe concentrations are associated with higher R_ct_ values. This hypothesis was confirmed by the experimental data in Table 3. The R_ct_ at 10^−20^ M *E*. *coli* DNA for a probe concentration of 32.5 nM was 1101 Ω, which decreased to 775.5 Ω and 542.7 Ω for lower probe concentrations of 3.25 nM and 0.325 nM, respectively.

**Table 3:** Fitting functions for the proposed sensors using various amine probe concentrations.

Probe concentration (nM)	Function	R^2^	R_ct_ (logC = -20 M) (Ω)
32.5	35.6 *x* + 1820	0.985	1101 ± 38.8
3.25	142 *x* + 3570	0.967	775.5 ± 42.1
0.325	172 *x* + 3990	0.999	542.7 ± 48.6

Furthermore, since the probe concentration is often a million times higher than the maximum concentration of target ssDNA in the analyte to ensure a uniform and sufficient probe layer for the complementary DNA to bind effectively, many excess probe molecules may remain on the sensor after DNA hybridization. These surplus probe molecules can hinder charge transfer and thus alter the detection signal. Reducing the number of redundant probes can enhance the detection signal by minimizing undesired charge resistance in the sensor, which may alter and disrupt the measured R_ct_ value. Hence, the steeper slopes at lower probe concentrations indicate increased analyte detection efficiency.

All the evidence presented above confirms that the proposed sensors are functional and possess a high potential for selectivity. To verify the performance of these sensors, we prepared six test samples with *E*. *coli* DNA concentrations ranging from 10^−19^ and 10^−17^ M for three types of sensors. The data acquisition and fitting process were repeated as described in previous sections. The results are summarized in Table 4. The calculated logC was derived based on the fitted R_ct_ values and the operating functions in Table 3. All calculated logC values are close to the tested values. The minor differences are less than 5%. Our study demonstrates that the proposed sensors can detect *E*. *coli* DNA from 10^−20^ to 10^-15^M with a limit of detection (LOD) of 10^-20^M. Notably, when compared to previous studies listed in Table 5, our sensors either exhibit a lower limit of detection or benefit from more cost-effective materials and simplified experimental procedures.

**Table 4:** Verification of proposed sensors with known concentration samples.

**32.5 nM Probe**			
**logC** ([C] = M)	**R**_**ct**_ (Ω)	**Estimated logC**	**Difference** (%)
-19	1155±39.6	-18.68	1.69
-17	1197±40.0	-17.50	2.94
**3.25 nM Probe**			
**logC** ([C] = M)	**R**_**ct**_ (Ω)	**Estimated logC**	**Difference** (%)
-19	870±61.6	-19.01	0.07
-17	1248±50.6	-16.35	3.81
**0.325 nM Probe**			
**logC** ([C] = M)	**R**_**ct**_ (Ω)	**Estimated logC**	**Difference** (%)
-19	715.7±45.8	-19.04	0.19
-17	1090±50.1	-16.86	0.82

**Table 5:** Comparison with other studies on detection techniques.

Biosensor material	Detection techniques	Linear range (M)	LOD (M)	Ref.
AuNPs and Cht-Au composite	Square wave voltammetry (SWV)	10^−19^ – 10^−14^ M (*stx1*), 10^−19^ – 10^−13^ M (*stx2*)	10^−19^	[28]
Ag/AgCl	Differential pulse voltammetry (DPV)	10^−19^–10^−10^	5.6×10^−19^	[29]
Hollow silica NPs	DPV	10^−18^–10^−8^	8.17×10^−20^	[30]
AuNPs and Cht-Au composite	SWV	10^−16^–10^−6^	10^−16^	[31]
Hybrid MoS_2_	EIS	10^−20^–10^−15^	10^−20^	This work

## Conclusions

In summary, this research introduces an application of new hybrid MoS2 nanosheets and microparticles. It successfully establishes the foundations for a robust *E*. *coli* sensing platform, leveraging impedance measurements and a combination of hybrid MoS_2_ materials and an amine probe. The proposed sensors exhibit linear functionality within a specified concentration range from 10^−20^ to 10^−15^ M, achieving an impressive detection limit of 10^−20^ M. Furthermore, the sensors’ distinguished selectivity underscores their potential, particularly in discerning *E*. *coli* DNA from that of *Bacillus subtilis* and *Vibrio proteolyticus*. This work sets a precedent for future innovations in the realm of DNA detection and paves the way for more sophisticated diagnostic tools.
